# The impact of COVID-19 and access to health services in the Hispanic/Mexican population living in the United States

**DOI:** 10.3389/fpubh.2022.977792

**Published:** 2022-11-25

**Authors:** María Gudelia Rangel Gómez, Jorge Alcocer Varela, Saúl Salazar Jiménez, Leonardo Olivares Marín, Cecilia Rosales

**Affiliations:** ^1^El Colegio de la Frontera Norte, Tijuana, Mexico; ^2^US-Mexico Border Health Commission, Tijuana, Mexico; ^3^Secretariat of Health in Mexico, Mexico City, Mexico; ^4^Division of Public Health Practice and Translational Research, Mel and Enid Zuckerman College of Public Health, University of Arizona, Phoenix, AZ, United States

**Keywords:** COVID-19, immigrants, Mexican, health, United States, diseases

## Abstract

**Introduction:**

The United States is home to 10.5 million undocumented immigrants, of which 5 out of 10 are Mexican or Central American. Their immigration status is an obstacle to secure employment that provides labor benefits such as sick leave and health insurance. Living through the global pandemic in the U.S. had a negative impact on this vulnerable population's mental and physical health. They avoided seeking primary or hospital care fearful that they were undocumented and uninsured. The services provided by the Ventanillas de Salud (VDS) “Health Windows” mitigated this pandemic's negative impact and have become an important source to support and increase access to health services among the immigrant community.

**Methods:**

De-identified data from a database system called the Continuous Information System and Health Reports of Mexicans in the United States (SICRESAL-MX) to perform this secondary analysis. The descriptive analysis describes socio-demographic, epidemiological, and situational characteristics of COVID-19.

**Results:**

Between January 2020 and July 2021, the VDS and UMS provided 11.5 million individual services to just over 4.3 million people. The main health conditions are overweight and obesity, high blood pressure and elevated cholesterol and glucose levels. Between March 2020 to July 2021 a total of 2,481,834 specific services related to COVID-19 were offered.

**Discussion:**

The Mexican migrant community in the United States is in a vulnerable situation, largely due to its immigration status which limits their access to health and human services, including primary health care services. Many of them have suffered from chronic diseases since before the pandemic, generating difficulties in monitoring the ailments and exacerbating their conditions.

## Introduction

The Hispanic immigrant population living in the United States face a series of social barriers that hinder their living conditions. Among them are the legal barriers stemming from their immigration status (1), which may result in difficult working conditions (2) and limited access to healthcare services, including basic health screens (3). Recent statistics based on data from the US Census Bureau (4), show that there are 10.5 million undocumented immigrants in the United States, of which approximately half come from Mexico and Central America.

The history of Mexican migration to the United States dates to the late 19th and early 20th centuries. At that time, the migratory dynamics were relatively unregulated, and the flow was almost natural from Mexico to the United States and vice versa. In the first decade of the 20th century, an agreement was signed between both governments for Mexican workers to work in US agriculture (5).

During World War I, the United States issued measures that sought to regulate migration in general. Under the Literacy Act, entry was conditioned to those who knew how to read and write, and could pay border crossing fees. During the economic crisis of 1929, the U.S. context showed greater hostility toward Mexican migration. Meanwhile, Mexico could not absorbed the growing demand for employment by the working-age population (5).

Beginning with World War II, the United States had a strong demand for labor in agriculture and other productive sectors due to the shortage of American workers. In 1942, the Bracero Program began, in which Mexico provided labor for agricultural production. Mexican migrant's wages were much lower and conditions more precarious than their U.S. counterparts. The program continued once the war was over until the United States ended it in 1964. From then on, migration to the United States was constant and growing, but mostly undocumented (5).

Mexican migratory flows to the United States maintained a growing trend in the 1980s and 1990s. As of 2001, due to the terrorist attacks, the context hardened through greater border control measures. The result was an increase in deportations and a large number of migrants who lost their lives trying to cross the border. By 2008, the repatriation of undocumented Mexican immigrants reached significant and alarming levels. Also, during this time, the Deferred Action for Childhood Arrivals (DACA) Program began, as a policy that serves and protects “Dreamers,” a population that had come to the United States as children, allowing them a legalized manner to remain in the United States (5).

The Donald Trump administration focused on an anti-immigrant discourse with the aim of building a wall on the northern border with Mexico (5). One of the initiatives, America First, consisted of increasing immigration restrictions in the name of protecting American workers and industry (6).

The immigrant population can be considered among the most vulnerable for several reasons. For example, an undocumented status precludes authorization to work legally in the country of residence which for many results in securing jobs that subjects them to low wages, long hours, and exposure to hazardous conditions (1) such as exposure to toxins, extreme temperatures, pesticides, and chemicals. Also, they may find employment in industries such as construction, agriculture, production, shipping, and transportation which are jobs known to have higher accidents rates compared to other types of jobs (2). In addition, undocumented status contributes to a web of interrelated consequences that can prevent immigrant workers from accessing the worker protections to which they are entitled.

Most Mexican immigrants have lower rates of health insurance coverage compared to the U.S. counterparts, resulting in limited use of emergency or primary care services, or accessing lower quality health care (3). Additional barriers to accessing services are limited English language proficiency, restrictive public health access policies, fear of deportation and discrimination, which can adversely affect communities (7).

The existence of mental health problems can contribute to vulnerability. The political environment in the U.S. increased anti-immigrant discourse and sentiment, attempts to tighten immigration laws, deportation threats, family separation, and detentions (8). In this context, the immigrant population has experienced fear, stress, anxiety, and perceptions of discrimination, thereby exacerbating mental and behavioral challenges (9, 10).

Scholarly literature has shown that high stress levels among individuals leads to disease susceptibility. Thus, living and working conditions contribute to poor health outcomes such as chronic diseases, including heart disease and diabetes (7, 11, 12). The immigrant population's situation worsened during the COVID-19 pandemic. The cumulative number of COVID-19 cases and hospitalizations in this community are among the highest in the country due in part to lack of access to healthcare (8, 13).

Therefore, immigration policies and laws play a central role in the quality of life of the immigrant population by shaping the type of employment and salary, the level of access to health services, as well as populations ailments (14). Immigration laws dictate who enters and who stays out, as well as the structural vulnerability of those who enter. The policies classify the population in different categories of precariousness, from illegality to temporary stay, permanent residence, and citizenship. This results in a differentiated labor supply that produces precarious workers (15, 16). Further, immigration laws require certain skills and experience for the different categories. This places various restrictions on the freedoms of the population, their privileges, and rights of those who enter the United States, which has an impact on their labor market insertion (17).

During the pandemic, immigrants were well-known as the nation's essential workers. They continued to work despite the risk for exposure to the novel coronavirus. For example, early in the pandemic, the agricultural sector failed to protect its workers by implementing recommended mitigation measures such as social distancing, handwashing and mask wearing; prior to the development of vaccines (18–20).

In the hotel and restaurant industry, immigrant workers lost employment. Travel restrictions and quarantine measures had a negative impact on this sector with the loss of jobs and financial security. While there has been discussion and concern about how this pandemic has affected travel and the hospitality industry, there has been less concern about the impact on the millions of essential workers that lost jobs in this industry (7). Overall, in the United States, 51 million jobs were lost during 2020 because of the pandemic, an all-time record (21).

Living in this context disempowers the immigrant population from seeking healthcare services, creating a danger for all during a global pandemic (22, 23), more so when care is inaccessible. For example, 38% of Mexican immigrants in the United States do not have health insurance, while 8% of those born in the United States are uninsured (24). Moreover, the working conditions, economic status and housing conditions of this sector may increase exposure to the coronavirus, as well as transmission and spread (8).

The Ventanillas de Salud (VDS), pre-COVID-19, have been shown to be an important and trusted source of information providing access to health services for the immigrant community since 2003 (25). They were established by the Health Secretariat and the Foreign Affairs Secretariat, through the Institute of Mexicans Abroad (Instituto de los Mexicanos en el Exterior). The VDS's mission is to improve access to primary and preventive healthcare services, increase public insurance coverage, connect people to medical homes, and promote a culture of self-care among Mexicans living in the United States (26).

In addition to offering general health information, the VDS provides counseling, health education, disease prevention, and health promotion. Also, they offer preventive health screens, referral to primary healthcare services, and health insurance navigation resources. The existing 49 VDS and two mobile VDS are housed within the Mexican consular network in the United States and operated by local agencies (27).

Likewise, in 2016, the Mexican Section of the United States-Mexico Border Health Commission (USMBHC) introduced the Mobile Health Units (MHU) model of care to strengthen the VDS programs. The objective of the MHU is to outreach and bring preventive health services to remote communities with difficult access to healthcare services, reaching the most vulnerable (3). Currently there are 11 MHU located in Chicago (IL), Dallas (TX), Los Angeles (CA), Phoenix and Tucson (AZ), NYC (NY), Denver (CO), Las Vegas (NV), Miami and Orlando (FL), and Raleigh (NC) (3, 28, 29).

As part of the same strategy, the VDS and MHU serve the same target population, Mexican migrants in the United States. The difference is that the VDS are located inside the offices of the General Consulates of Mexico in 49 U.S. cities. While MHU serve the population that is hard to reach; due to distance, lack of transportation, resources, or fear due to their immigration status (30). In summary, while the VDS receives the population, the UMS goes to where the people are.

Although the VDS and MHU were initially developed to address the needs of the Mexican immigrant population, they serve all those in need, regardless of their country of origin or immigration status.

The objective of this article is to describe the socio-demographic and epidemiological characteristics of the Latino immigrant population served in the VDS and MHU living in the United States, from January 2020 to July 2021. It also seeks to describe the impact and situation of the target population in the face of the COVID-19 pandemic.

## Methods

The authors used de-identified data from a database system called the Continuous Information System and Health Reports of Mexicans in the United States (SICRESAL-MX [acronym in Spanish]) to perform this secondary analysis (31). SICRESAL-MX is a computer-based system developed by the Mexican Section of the USMBHC, specifically to confidentially maintain information provided by users in the VDS and MHU. The descriptive analysis describes socio-demographic, epidemiological, and situational characteristics of COVID-19. Use of secondary data for this analysis was not deemed human subjects research, therefore, did not require IRB approval.

## Results

### Socio-demographic analysis

From January 2020 to July 31, 2021, the VDS and MHU provided a total of 11.5 million individual services to 4.3 million individuals. These services included counseling, education, COVID-19 and Influenza vaccination, basic health screening, and referrals (Table 1).

**Table 1:** Individual services provided in VDS and UMS by type of service, January 2020–July 2021.

**Variable**	**Frequency**
Population served	**4,267,808**
Individual services provided	**11,475,047**
Guidance and education	10,429,503
Vaccination	230,437
Basic Health Screening	577,818
Referral	13,079

Source: Prepared by the authors based on SICRESAL-MX, Mexican Section of the United States-Mexico Border Health Commission. The numbers in bold are to highlight the total numbers of population served and services offered (the figures that are not in bold when added together make up the total services offered).

Sixty percent (2'5617,153) service users are female and slightly more than half (56%, 2'389,972) are between the ages of 30 and 49. Among those under 18 years of age (7%, 298,747) and older adults aged 60 years or older (9%, 384,102) use these services (Table 2). Half of the users reported attaining a middle school education (50.1%, 2,138,172) and a 34% (1,468,126) reported having attended between the ninth and twelfth grade; 7.2% (307,282) did not complete college and 6.8% (290,211) completed college.

**Table 2:** Main socio-demographic characteristics of the population served at VDS and MHU, January 2020-July 2021^*^ *N* = 4,267,808.

**Characteristics**	**Percent**	**Characteristics**	**Percent**
**Gender**	**100**	**English level**	**100**
		Basic	13.9
Male	40.9	Intermediate	50.8
Female	59.0	Advanced	35.3
Transgender	0.1		
**Age group**	**100**	**Education level**	**100**
Under 10	3.8	None	1.5
10–14	0.7	1–6	28.8
15–19	2.0	7–9	21.3
20–29	10.8	9–12	34.4
30–39	24.9	Some years of college	7.2
40–49	31.3	University/College	6.8
50–59	17.4		
60 and more	9.1	**Occupation**	**100**
**Birthplace**	**100**	Cleaning services	23.7
Mexico	94.6	Construction	22.1
USA	2.9	Manufacturing/Factories	14.1
Central America	1.6	Cook/Bartender/Waiter	10.6
South America	0.7	Gardening/Agriculture	8.3
Caribbean	0.2	Administrative	8.1
**Years living in the United States**	**100**	Sales	3.4
Under 1 year	2.7	Driver	2.4
1–4 years	8.0	Other occupations	7.3
5–9 years	9.8		
10 years or more	79.5		

Source: Prepared by the authors based on SICRESAL-MX, Mexican Section of the United States-Mexico Border Health Commission.

About 80% (3'414,246) of the users of these programs are permanent residents, having lived in the U.S. for 10 years or more. Another 10% (426,781) have lived in the U.S. 5–9 years, and an additional 10% (426,780) have recently migrated within 4 years or less.

Regarding the place of birth, 94.6% (4,037,346) of the population served is of Mexican origin. Although this population is the objective, the VDS also provided service to other nationalities of which 2.9% (123,766) are Americans and between Central America, South America and the Caribbean make up the remaining 2.5% (106,695).

The level of English proficiency declared by the population served is mostly intermediate with 50.8% (2,168,046). 35.32% (1,506,536) stated that their level of English is advanced, while the remaining 13.85% (593,225) stated that their level is basic. Regarding occupations, the three main ones are cleaning services (23.7%, 1,011,470), construction (22.1%, 943,186) and manufacturing or factories (14.1%, 601,761); which make up about 60% of the occupations of the population served. The remaining 40% (1,711,391) is made up mostly of waiters, agriculture or gardening, administrative positions, sales, and drivers.

### Main conditions

The analysis of the epidemiological characteristics of the population allows us to know the main prevalence of diseases detected in the population attended in the VDS and MHU. The screenings made were about glucose, overweight and obesity, blood pressure, cholesterol, sexually transmitted infections (STI), and HIV. Although the relationship between COVID-19 and its associated conditions has not yet been examined, according to the Pan American Health Organization (32), people with chronic non-communicable diseases have a higher risk factor for complications from a COVID-19 infection.

The care provided to Mexican immigrants in the U.S. detected the main health conditions using various measurements (see Table 3). Seventy-eight percent (4,830) of the users of these services were overweight and obese. Thirty-seven percent (2,625) had high blood pressure, 31% (444) had elevated cholesterol levels, and 24.6% (1,376) elevated glucose levels. Other self-reported conditions included sexually transmitted infections (STI) at 1% (1) and < 1% percent reporting HIV (Acquired Immunodeficiency Virus) infection. In addition to the health screenings, guidance and education services were provided on care and prevention of these conditions. Most notable is the high uninsured rate at 97% (4,139,774).

**Table 3:** Prevalence of the main health conditions treated at the VDS and MHU, January 2020–July 2021.

**Type of detection**	**Guidance/Education provided**	**Measurements performed**	**Elevated readings detected**	**Prevalence%**
Glucose	10,927	5,594	1,376	25
Overweight and obesity	9,154	6,280	4,830	77
Blood Pressure	11,527	7,013	2,625	37
Cholesterol	9,154	1,430	444	31
STI	3,241	360	4	1
HIV	3,241	402	2	1

(^1^) Includes guidance and education on obesity/metabolic syndrome/cholesterol prevention.

(^2^) Includes guidance and education on HIV and STI prevention.

Source: Prepared by the authors based on SICRESAL-MX, Mexican Section of the United States-Mexico Border Health Commission.

### Situational diagnosis of COVID-19

In response to the pandemic, the VDS and MHU programs included COVID-19 in their guidance, education, health screening, primary care referrals, and vaccination services. During the “Stay at Home” orders across the U.S., staff adapted and continued to provide services remotely from their newly created “home offices” through phone calls, e-mails and social networks. Upon lifting the shelter in place orders, staff returned to the community, adjusting their approach to implement the CDC recommendations of physical/social distancing, hand sanitizing and mask wearing. Once vaccines became available in 2021, with collaboration with local health departments, staff included COVID-19 and Influenza vaccination services.

As a result, from March 2020 to July 31, 2021, the VDS and MHU offered a total of 2,481,834 specific services related to COVID-19. The actions carried out focused on guidance and education (1.47 million), dissemination of credible and evidenced-based information on COVID-19 on social networks (699,000), in addition to health screenings (157,000) and vaccination (156,000).

### Impact of COVID-19

The following describes the social impact of the pandemic on individual users of the VDS and MHU, their families, and friends (Table 4). Of all users of the VDS and MHU during COVID-19, 56% (8,695) were women, 6 of every 10 (12,250) users were between 31 and 50 years of age. Remarkably, only 3% (605) of all services users were 60 years of age or older.

**Table 4:** COVID-19 situational diagnosis of population served in VDS and MHU, March 2020 to July 2021^*^ *N* = 19,929.

**Variables**	**Percent**	**Variables**	**Percent**	**Variables**	**Percent**
**Gender**	**100**	**Have experienced**	**100**	**Lost their job due to the COVID-19 outbreak**	**15.0**
Male	43.6	Food insecurity	47.4		
Female	56.3	Lack of hygiene products	15.1	**Have a relative unemployed due to the COVID-19 outbreak**	**20.5**
Transgender	0.1	Loss of income	28.6		
**Main source of information about the pandemic**	**100**	Other	8.9		
		**Tested for COVID-19**	**32**	**Sought assistance of a government program**	**6**
T.V.	60.7	Positive	22		
Social media	21.9	Negative	73	**Sought assistance of a food bank**	**20**
Friends/Relatives	8.1	**Symptoms experienced**	**100**		
Radio	4.6	Dry cough	13.9	**How serious do you think the current situation is?**	**100**
Other	4.7	Headache	13.4		
**Level of information you have regarding the pandemic**	**100**	Body pain	12.9	Very serious	38.2
		Sore throat	12.8	Serious	54.3
Very good	31.0	Fever	11.4	I don't believe it exists	0.8
Good	60.5	Other	35.6	Not serious	6.7
Regular	7.8	**With someone you know diagnosed with COVID-19**	**32**		
Uninformed	0.7				

Source: Prepared by the authors based on SICRESAL-MX, Mexican Section of the United States-Mexico Border Health Commission. The letters in bold correspond to each question asked to the population served. Those not in bold are the answers that make up the question. For example: Gender - 100%; Male - 43.6%, Female - 56.3%, Transgender - 0.1%. There are questions that have no answers, for example: Lost their job due to the COVID-19 outbreak - 15% (it means that 15% of the population served lost their job due to the pandemic).

Among the immigrant population seeking advice, education and guidance, 60% (12,101) indicated their main source of information about the current pandemic was television followed by social media. The majority reported they had a “Good” or “Very Good” level of information.

At least 6% (1,164) of the users who approached the VDS or MHU reported symptoms related to COVID-19. The most common symptom dry cough, manifested in 14% (2,769), followed by headache (13%, 2,661), body aches (13%, 2,562) and sore throat (13%, 2,543), fever above 98.6°F/ 37°C (11%, 2,265), chest pain (10%, 2,015) and joint pain (9%, 1,882), among others. One out of 10 (2,192) reported they required a hospital or clinic visit due to symptoms related to COVID-19 and 32% (6,377) had been tested for coronavirus, of which 22% (1,372) tested positive.

Between January and July 2021, the VDS and MHU administered vaccinations against COVID-19. From a total of 12,913 people served, 34% (4,390) reported having received a COVID-19 vaccine, the remaining hesitated to receive the vaccine. The main reason for not receiving the vaccine was distrust in the government (28%, 2,434). For this same period, 90% (18,431) of the users considered the current pandemic as serious or very serious, yet reported not receiving the COVID-19 vaccine.

The VDS and MHU users' perception of how serious the current situation is, relates to the social impact in their own lives. For example, 32% (6,285) of the users reported a member of their family, friend, co-worker, or neighbor tested positive for COVID-19; 21% (4,088) stated a member of their family experienced a job loss due to the pandemic, and 15% (2,926) reported personal job loss. Economically, 47% (9,455) stated that during this period, they experienced food insecurity, and 29% (5,691) experienced loss of income. Consequently, 20% (3,970) of the users indicated they required food bank services, while 6% (1,197) of the total made use of one of the programs launched by the city, county, or state governments to support the community with the challenges faced.

In addition, the emotional health of users has also been affected in different ways: 29% (5,684) expressed stress, 22% (4,345) concern about their future, 21% (4,111) anxiety, 11% (2,251) felt isolated, 11% (2,116) tired, and 7% (1,423) sadness or loneliness.

## Discussion

The Mexican migrant community in the United States is in a vulnerable situation, largely due to its immigration status which limits their access to health and human services, including primary health care services. Many of them have suffered from chronic diseases since before the pandemic, generating difficulties in monitoring the ailments and exacerbating their conditions. The main conditions that affect the population studied are overweight and obesity, high blood pressure, high cholesterol, and high glucose levels while almost entirely lacking health insurance.

Social factors that hinder further access to health services are low levels of formal educations, a limited command of the English language, and precarious jobs, which translates into the quality of life of this population. In addition to the above, the COVID-19 pandemic had an impact on their vulnerability, reflected in job losses, food shortages, and income loss, leading some to request assistance from food banks or government aid while also affecting their mental health, experiencing higher levels of stress and worry about the future.

Prior to the current pandemic, the VDS and MHU were trusted voices and sources of care, providing basic health screenings, health education, and referral to primary care. Both were even more essential in enhancing the reach of local health departments in providing COVID-19 testing, mitigation guidance, and COVID-19 vaccination, when it became available. Reaching this population through the VDS and MHU is a practical option for receiving guidance and education services provided in Spanish, the native language of most. Nonetheless, outreach and primary prevention services cannot replace the much-needed primary care that many VDS and MHU users require. Therefore, the challenges to serving the migrant community require the coordination and cooperation of both Mexican and U.S. agencies.

## Conclusion

Keeping the migrant community informed and aware of available resources, educating and assisting them in navigating the healthcare and public healthcare system in the U.S. is challenging under normal or non-crisis circumstances, let alone during what we have experienced globally with the current pandemic. Migrant communities are fluid and require consistent and reliable engagement, providing credible, culturally sensitive information, and cultural humility. Moreover, coordination and cooperation between and among trusted community-based organizations, community leaders, and local/state health authorities is critical in creating community well-being.

The analyzed results aim to demonstrate the action capacity of the VDS and MHU. Given the success they have had with the Latino migrant community in the United States, this strategy can be a model applicable to other organizations dedicated to the care of vulnerable groups. The strategy was able to adapt to the emergence of health emergencies and has shown that it can be part of a response protocol in the event of a health emergency, in this case, the COVID-19 pandemic. Finally, this strategy contributes to access to first level health services since these cannot influence the health strategies of the US government.

Primary health care has had a very good impact on the Latino immigrant community in the United States. Primary health care, being free regardless of immigration status, has made it possible to care for an entire population that would otherwise be very difficult or had no access. The limitations of the strategy are imposed by the various contexts and challenges they present. Each VDS and MHU, the agencies that work for them, and their allies face a variety of situations that vary from city to city.

## Data availability statement

The original contributions presented in the study are included in the article/supplementary material, further inquiries can be directed to the corresponding author/s.

## Author contributions

SS and LO contributed to the writing and initial data analysis. MR, JA, and CR contributed to the data analysis review, discussion, and data interpretation. All the authors contributed to the article and approved the version submitted.

## Funding

The initial funding for this project was provided by the Mexican Government.

## Conflict of interest

The authors declare that the research was conducted in the absence of any commercial or financial relationships that could be construed as a potential conflict of interest.

## Publisher's note

All claims expressed in this article are solely those of the authors and do not necessarily represent those of their affiliated organizations, or those of the publisher, the editors and the reviewers. Any product that may be evaluated in this article, or claim that may be made by its manufacturer, is not guaranteed or endorsed by the publisher.
